# Collagen Inhibitory Peptide R1R2 Mediates Vascular Remodeling by Decreasing Inflammation and Smooth Muscle Cell Activation

**DOI:** 10.1371/journal.pone.0117356

**Published:** 2015-02-12

**Authors:** Ting-Hein Lee, Jane Sottile, Hou-Yu Chiang

**Affiliations:** 1 Department of Anatomy, College of Medicine, Chang Gung University, Tao-Yuan, Taiwan; 2 Aab Cardiovascular Research Institute, University of Rochester School of Medicine and Dentistry, Rochester, New York, United States of America; INSERM, FRANCE

## Abstract

The extracellular matrix (ECM) is a major constituent of the vessel wall. In addition to providing a structural scaffold, the ECM controls numerous cellular functions in both physiologic and pathologic settings. Vascular remodeling occurs after injury and is characterized by endothelial cell activation, inflammatory cell infiltration, phenotypic modulation of smooth muscle cells (SMCs), and augmented deposition of collagen-rich ECM. R1R2, a peptide derived from the bacterial adhesin SFS, with sequence homology to collagen, is known to inhibit collagen type I deposition in vitro by inhibiting the binding of fibronectin to collagen. However, the inhibitory effects of R1R2 during vascular remodeling have not been explored. We periadventitially delivered R1R2 to carotid arteries using pluronic gel in a vascular remodeling mouse model induced by blood flow cessation, and evaluated its effects on intima-media thickening, ECM deposition, SMC activation, and inflammatory cell infiltration. Morphometric analysis demonstrated that R1R2 reduced intima-media thickening compared to the control groups. R1R2 treatment also decreased collagen type I deposition in the vessel wall, and maintained SMC in the contractile phenotype. Interestingly, R1R2 dramatically reduced inflammatory cell infiltration into the vessel by ∼78%. This decrease was accompanied by decreased VCAM-1 and ICAM-1 expression. Our in vitro studies revealed that R1R2 attenuated SMC proliferation and migration, and also decreased monocyte adhesion and transendothelial migration through endothelial cells. Together, these data suggest that R1R2 attenuates vascular remodeling responses by decreasing inflammation and by modulating SMC proliferation and migration, and suggest that the R1R2 peptide may have therapeutic potential in treating occlusive vascular diseases.

## Introduction

The extracellular matrix (ECM) is a major component of tissues. In addition to providing structural support for tissues, the ECM is also an important regulator of cell proliferation, migration and differentiation. The composition and organization of ECM proteins is dynamically remodeled by cell-mediated regulation of ECM synthesis, deposition, degradation, and the interplay between ECM proteins. Tight regulation of ECM remodeling is essential for maintaining normal physiological processes, such as development and wound healing [1–4]. In contrast, dysregulation of ECM remodeling can disrupt tissue homeostasis and result in pathological conditions. In addition to inflammatory cell infiltration and smooth muscle cell activation, ECM remodeling is one of the hallmarks of pathologic vascular remodeling [4–8]. Changes in the ECM can have profound impacts on vascular architecture and cellular behavior [9–12]. Collagen type I is the most abundant ECM protein in the vessel wall, and augmented collagen type I accumulation is observed in vessels following injury [7,13,14]. In vivo studies demonstrate that excess collagen type I deposition in the vessel wall changes the collagen/elastin ratio and alters the mechanics of the blood vessels, resulting in increased arterial stiffness [15–17]. Furthermore, by binding to cell surface receptors such as integrins or discoidin domain receptors (DDRs), collagen type I acts as a signaling molecule which affects vascular endothelial and smooth muscle cell growth and migration [18–22]. Therefore, methods that manipulate ECM remodeling and efficiently decrease collagen type I deposition in the vessel wall have the therapeutic potential to interrupt the progression of occlusive vascular diseases.

The close relationship between fibronectin (FN) and collagen has long been described. FN can bind directly to collagen type I with a well-characterized collagen-binding domain [23–25]. Extensive co-distribution of FN with collagen type I is observed in tissues as well as in cultured cells [26,27]. In vitro studies have shown that deposition of collagen type I fibrils is dependent on the presence of FN matrix [28–31]. Inhibiting FN-collagen binding impairs collagen fibril formation around cells and decreases SMC migration [29]. In addition, previous studies have revealed that repression of collagen expression attenuates neointimal formation in an animal model [32]. In this study, we used the peptide, R1R2, which is known to inhibit collagen type I deposition by blocking FN-collagen binding [33], to address the role that collagen type I deposition plays during vascular remodeling. The R1R2 peptide is derived from a FN binding bacterial adhesion, and contains a sequence similar to sequences in types I-IV collagen [33]. We delivered R1R2 periadventitially using pluronic gel in a flow-induced vascular remodeling model in mice [34,35] and assessed its effects on the extent of neointimal formation, SMC activation, and inflammatory cell infiltration. Our results show that R1R2 attenuates vascular remodeling by decreasing early inflammatory cell invasion and SMC phenotypic modulation.

## Materials and Methods

### Materials

The R1R2 and scrambled peptides were custom synthesized by Kelowna International Scientific Inc. (Taipei, Taiwan). The sequence of R1R2 used in this study is: GLNGENQKEPEQGERGEAGPPLSGLSGNNQGRPSLPGLNGENQKEPEQGERGEAGPP. The scrambled peptide is: PGPGAEQPEQSKERNSQERGNGLALPGEELEGQEGGNKPSGENNGPPQGNLRGPLEG. Human FN and rat collagen type I for competitive ELISA were purchased from Sigma-Aldrich (St. Louis, MO). TNF-α for in vitro assays was from Roche (Basel, Switzerland).

### Cell Culture

Human umbilical vein endothelial cells (HUVECs) and A7r5 SMCs were provided by Resource Center (SB3, NSC 100-2325-B-080-001), National Research Program for Biopharmaceuticals (Hsinchu, Taiwan). HUVECs were maintained in M199 (Life Technologies, Carlsbad, CA) with 25U/ml heparin (Sigma-Aldrich, St. Louis, MO), 10% fetal bovine serum (FBS) and endothelial cell growth supplement (ECGS; Millipore, Billerica, MA). A7r5 SMCs were grown in Dulbecco’s Modified Eagle’s Medium (DMEM) containing high glucose, supplemented with 10% fetal bovine serum. Human U937 cells were grown in RPMI1640 with 10% FBS.

### Enzyme-Linked Immunosorbant Assay (ELISA)

96-well plates were coated with 10 μg/ml collagen I or BSA at 4°C overnight. In separate plates, FN (4 nM) was incubated with the indicated amount of R1R2 or scrambled peptide at 4°C overnight. The FN-peptide solutions were added to collagen or BSA-coated wells, and the plates were incubated overnight at 37°C. The wells were washed and a rabbit anti-FN antibody was added for 90 minutes at room temperature. Wells were washed and then incubated with a horseradish peroxidase-conjugated secondary antibody. After washing was completed, peroxidase activity was quantified using 2,2'-azino-bis-(3-ethylbenthiazoline-6-sulfonic acid). Measurements were done at 405 nm (A405) using a SpectraMax M5 microplate reader (Molecular Device, Sunnyvale, CA).

### Ethics Statement

All animals and procedures in this study were approved by the Chang Gung University Institutional Animal Care and Use Committee (IACUC Approval No: CGU 10-025).

### Complete Common Carotid Ligation

6–8 week-old FVB/NJ (FVB) mice were subjected to complete common carotid ligation on the left side [34–36]. FVB mice are known to be very responsive to flow-induced vascular remodeling [37,38]. Animals were anesthetized with isoflurane inhalation. The left common carotid artery was exposed, dissected free from the surrounding connective tissue and completely ligated with 6-0 silk suture just proximal to the carotid bifurcation. In some animals, 50 μl of F-127 pluronic gel (BASF) with or without peptide was applied to the carotid artery periadventitially immediately after the ligation. 5 groups were used in this study: (1) sham-operated, in which the left carotid artery was exposed and dissected out, but not ligated (2) ligation (3) ligation with pluronic gel (4) ligation with pluronic gel containing 20 μM scrambled peptide (5) ligation with pluronic gel containing 20μM R1R2. 4–10 mice were included in each experimental group.

### Tissue Collection and Morphometric Analysis

5 groups of animals were processed for morphometric analysis. Mice were fixed with 10% formalin 7 or 14 days after the surgery. The common carotid arteries were harvested and embedded in paraffin. For each animal, cross-sections with 5μm thickness within the first mm proximal to the ligature were stained with Verhoeff-van Gieson elastic stain then the lumenal, intimal+medial area, and the area included within the external elastic lamina (EEL) were analyzed with Image-Pro Plus software (Media Cybernetics, MD) as described [35,36].

### Immunocytochemistry and Quantitative Analysis

Paraffin sections selected from the first mm of the carotid artery in each group were used for immunohistochemistry (IHC). The primary antibodies used were: polyclonal anti-collagen I (Millipore, Billerica, MA), polyclonal anti-FN (Millipore, Billerica, MA), polyclonal anti-smooth muscle myosin heavy chain (SM-MHC) (Biomedical Technologies, Stoughton, MA), monoclonal anti α-smooth muscle actin (Sigma-Aldrich, St. Louis, MO), polyclonal anti-Ki-67 (Abcam, Cambridge, UK), anti-leukocyte common antigen, CD45 (BD Pharmingen, San Jose, CA), monoclonal anti-intercellular adhesion molecule-1 (ICAM-1) (BD Pharmingen, San Jose, CA) and polyclonal anti-vascular cell adhesion molecule-1 (VCAM-1) (Santa Cruz Biotechnology, Dallas, Texas). Sections were then incubated with appropriate biotinylated secondary antibodies followed by avidin-biotin immunoperoxidase system (Vector Laboratories, Burlingame, CA). Liquid DAB Substrate Chromogen system (Dako, Carpinteria, CA) was used for detection.

Quantitative IHC analysis was performed on digital images captured with a 20x objective by using Image-Pro Plus software. Data from 3–6 mice for each group were averaged, and the average values ± SEM were shown. For FN, collagen type I, SM-MHC and SM α-actin, color digital images were transformed into gray scale, and the sum of the optical densities from the intima+media area in each section was determined. For evaluation of proliferating cells, the percent of Ki-67 (+) cells to total cells was determined by counting cell numbers in the immunostained sections that had been counterstained with hematoxylin. The percent of positive cells is reported as the proliferation index. For quantitative evaluation of leukocyte infiltration and cell adhesion molecule expression, the positive staining area for CD 45, ICAM-1 and VCAM-1 were obtained using an automated programmed segmentation procedure in Image-Pro Plus software. The intima+media region was traced manually. The percent of positively stained area in intima+media to the total traced area was assessed.

### Western Blot Analysis

Tissues or cells were homogenized with RIPA buffer [39] supplemented with protease inhibitor cocktail (Sigma-Aldrich, St. Louis, MO). Cell lysates were spun at 12,000 rpm for 15 min at 4°C, then electrophoresed on 10% SDS-PAGE gel and transferred to nitrocellulose. The membranes were incubated with antibody to collagen type I (1:1000; Millipore, Billerica, MA), FN (1:8000; Sigma-Aldrich, St. Louis, MO), ICAM-1 (1:500; GeneTex, Irvine, CA), VCAM-1 (1:1000; Santa Cruz Biotechnology, Dallas, Texas), or GAPDH (1:5000, Sigma-Aldrich, St. Louis, MO) overnight, followed by horseradish peroxidase–conjugated secondary antibodies (1:12000; BioRad, Hercules, CA) for 1 h at room temperature. Blots were developed using enhanced chemiluminescence (Thermo Scientific, Rockford, IL).

### Hydroxyproline Assay

In each independent experiment, three common carotid arteries 14 days after ligation per group were pooled, homogenized, precipitated with trichloroacetic acid, and baked overnight at 110°C in 37% hydrochloric acid. After reconstituting with water, samples were measured using a colorimetric chloramine T assay [40]. Three independent experiments were performed for statistical analysis.

### SMC Migration Assay

A scrape wound assay was used to assess the effect of R1R2 and scrambled peptides on SMC migration as described [36]. Briefly, A7r5 SMCs were grown until 90% confluent, followed by serum starvation and treatment with R1R2 and scrambled peptides for 36 hours. Several scrape wounds were then performed across the dish with a sterile yellow pipet tip. We initiated the experiment with treatment of cells with 10 ng/ml PDGF-BB. Photographs of the wound area were taken immediately after PDGF-BB addition (0 hour) and 30 hours postwounding with an Olympus CKX-41 microscope connected to a camera. At least three photographs were taken per group at each time point. Wound areas were determined using Image-Pro Software (Media Cybernetics, MD). Measurement of migration was determined by subtracting the cell-free area 30 hours after PDGF-BB addition from the cell-free area in the beginning of scrape wounds.

### Cell Proliferation Assay

A7r5 cells and HUVECs were growth-arrested and then stimulated with 10% FBS or 10 ng/ml TNF-αovernight after serum starvation as indicated in the figure legend. Briefly, trypan blue-excluding cells were counted with a hemocytometer, and data were analyzed using GraphPad Prism Software (San Diego, CA) as in previous studies [36]. A second observer performed a similar analysis to verify the initial findings.

### Monocyte Adhesion to HUVECs

HUVECs were cultured on gelatin-coated 24-well plates and pre-treated with 10 ng/ml TNF-α (Roche, Basel, Switzerland) for 6 hours after serum starvation. Monocyte U937 cells were stained with calcein-AM and added onto HUVECs at a concentration of 5x10^5^ cells/well for 30 minutes at 37°C. The media were gently removed, and the cells were washed three times with PBS. The number of U937 that were adherent to HUVEC was quantitated by the fluorescence of calcein-AM using a SpectraMax M5 microplate reader (Molecular Device, Sunnyvale, CA) set at 485 (excitation)/535 (emission). The number of cells per cm^2^ was calculated in comparison with the fluorescent intensity derived from known cell numbers of labeled U937 cells.

### Monocyte Transendothelial Migration Assay

HUVECs were grown on gelatin-coated 5um pore-sized transwells in 24-well plates (Corning, Corning, NY) and pre-treated with 10 ng/ml TNF-α (Roche, Basel, Switzerland) overnight after serum starvation. Briefly, calcein-AM stained U937 cells were added onto HUVECs at a concentration of 1.5x10^5^ cells/transwell for 1.5 hours at 37°C. After aspirating the media from the top well and washing with PBS three times, the transwells were transferred into new 24-well plates containing 800 ul cell dissociation buffer and cells were incubated for 1 hour at 37°C in a CO2 incubator to detach the transmigrated cells into the buffer. Transmigrated U937 were quantitated by the fluorescence intensity of calcein-AM using a SpectraMax M5 microplate reader (Molecular Device, Sunnyvale, CA) set at 485 (excitation)/535 (emission). The number of cells per well was calculated in comparison to fluorescent intensity derived from known cell numbers of labeled U937 cells.

### Statistics

For in vivo experiments, the data are shown as the mean ± SEM. For in vitro experiments, the data shown are the means ± SD of a representative experiment, at least 3 independent experiments were performed. One-way ANOVA with post-hoc testing were used for data analysis with GraphPad Prism software (GraphPad, San Diego, CA). If normality test failed, nonparametric Kruskal-Wallis test followed by Dunn's post-hoc test were used. P > 0.05 was not considered significant.

## Results

### Collagen Inhibitory Peptide R1R2 Blocks the Binding of Collagen to FN

**Fig 1:**
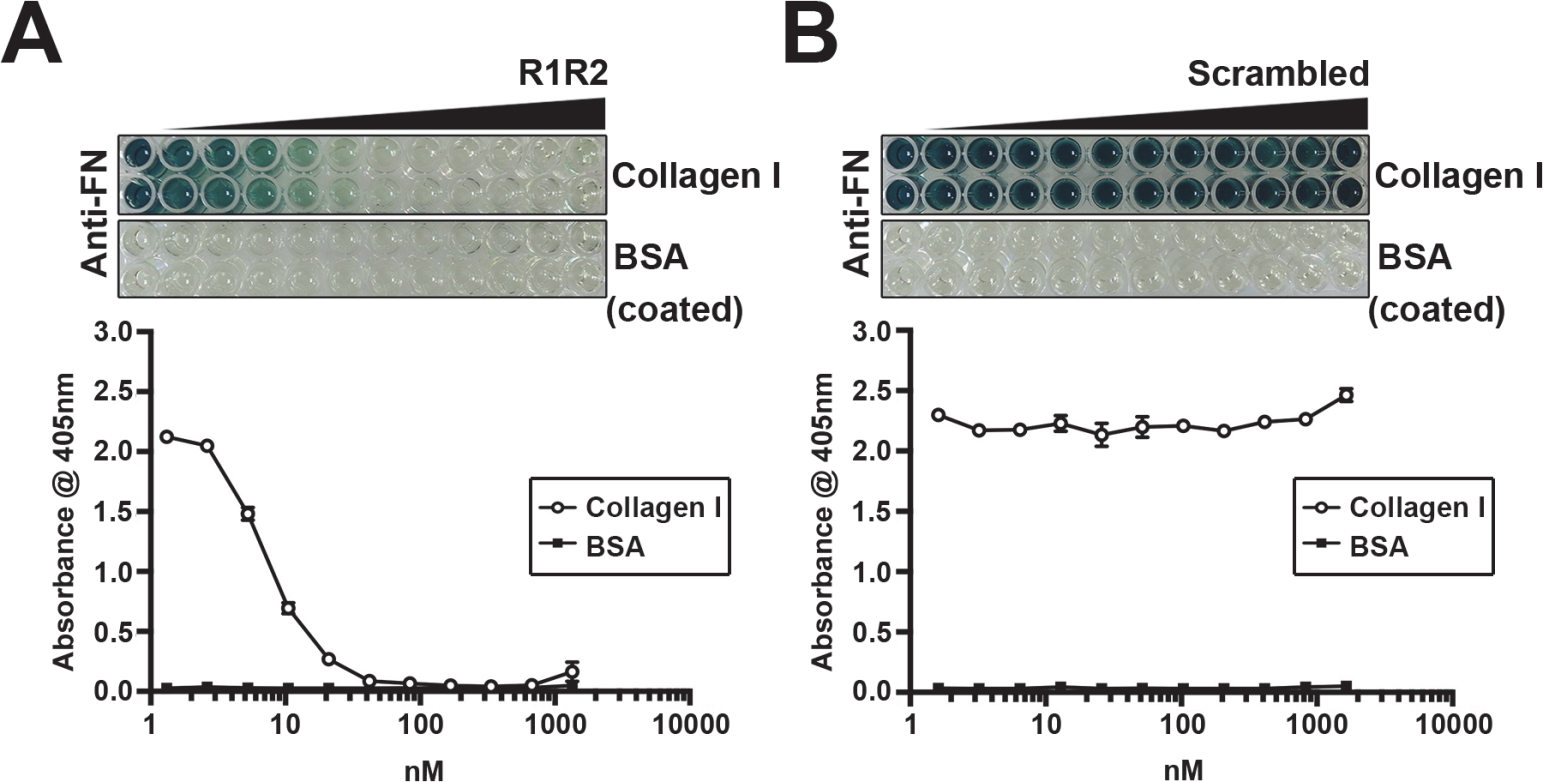
R1R2 decreases the binding between fibronectin (FN) and collagen. 4 nM of FN was incubated with the indicated amount of R1R2 or scrambled peptide at 4°C overnight. The mixture of FN and R1R2 or scrambled peptide was added into collagen or BSA coated wells. Bound FN was detected with an anti-FN antibody followed by HRP-conjugated secondary antibody, and ABTS as described in Methods. The absorbance at 405nm was quantitated.

In order to assess whether inhibiting collagen type I deposition reduces vascular remodeling in an in vivo mouse model, we used the collagen inhibitory peptide R1R2 for this study. The R1R2 peptide is derived from two repeat domains of the bacterial adhesin SFS, and is highly homologous to collagen. R1R2 is known to inhibit collagen-FN binding, collagen type I deposition, and smooth muscle cell migration in vitro [29,30,33]. It is likely that R1R2 blocks collagen type I fibril formation around cells by binding to the collagen-binding domain of FN. To characterize the effects of our synthesized R1R2, and a newly generated scrambled peptide, we performed a solid-phase binding assay to determine the effect of R1R2 on FN binding to collagen. As shown in Fig. 1, R1R2 blocked the binding of FN to collagen type I in a dose-dependent manner. In contrast, the scrambled peptide had no effect on FN-collagen binding. Therefore, we used our synthesized R1R2 and scrambled peptides for further experiments in this study.

### R1R2 Attenuates Neointimal Hyperplasia

**Fig 2:**
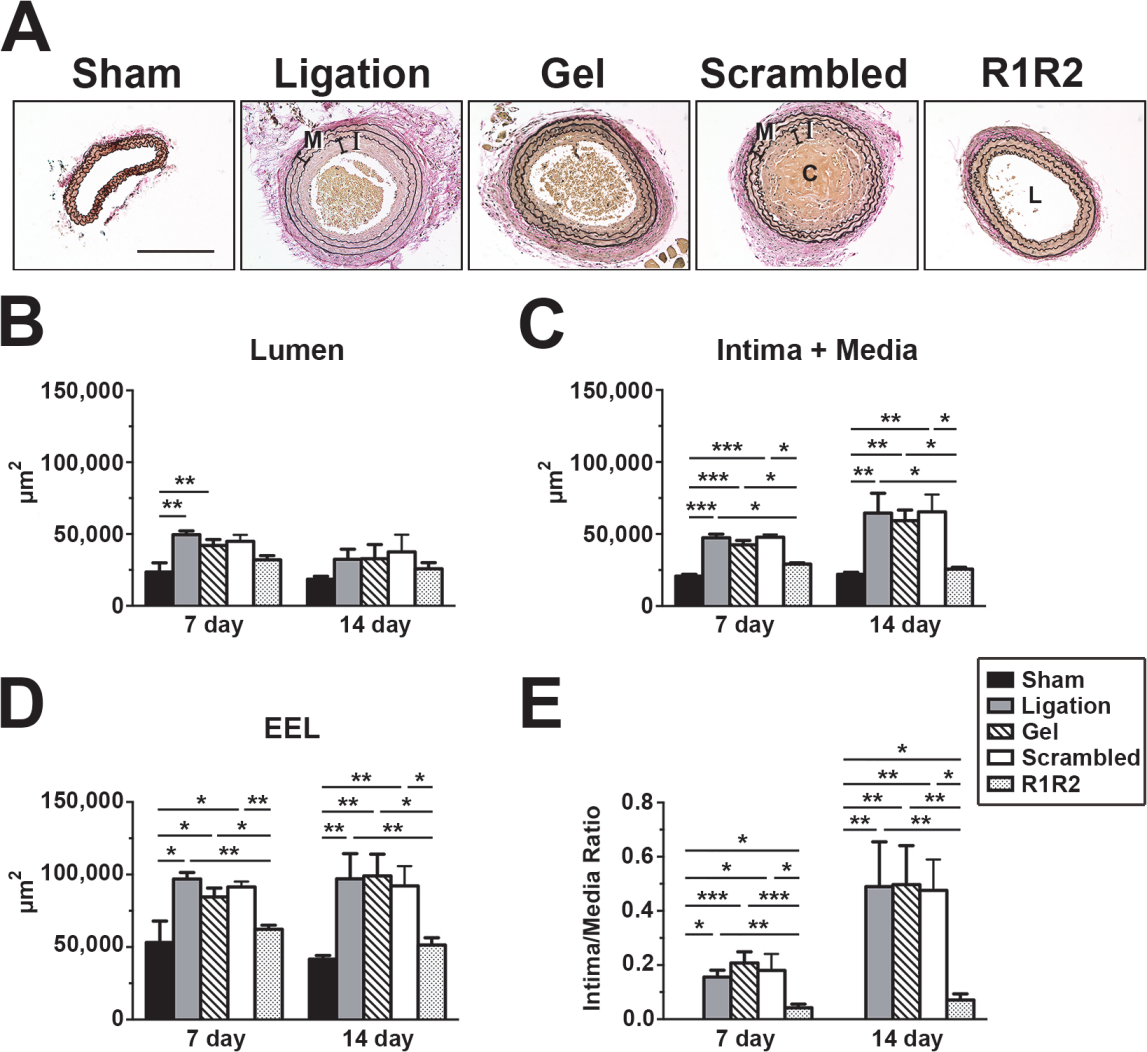
The collagen homologous peptide, R1R2, decreases vascular remodeling in the carotid artery. (A) Representative photomicrographs of the left carotid artery 14 days after ligation. Lumen (L), neointima (I) and media (M) in ligated vessels are shown. C indicates blood clot. Bar, 200 μm. Morphometric analyses of the lumen (B), intima+media (C), the external elastic lamina compartment (EEL; D), and intima/media ratio (E) 7 and 14 days after ligation. * indicates *p*<0.05, ** *p*< 0.01 and *** *p*< 0.001.

To directly test the effect of the R1R2 peptide in a model of vascular remodeling in mice, we evaluated the extent of neointimal formation in the presence or absence of the R1R2 peptide 7 days and 14 days after completely ligating the left common carotid artery in FVB mice. In this flow-induced model of vascular remodeling, neointimal formation is accompanied by significant leukocyte infiltration, proliferation of VSMCs, and extensive ECM accumulation; the endothelium remains intact [34–36]. R1R2 was periadventitially applied into carotid arteries using pluronic gel. We previously showed that a recombinant peptide can be delivered periadventitially by pluronic gel into the intima and media layers of mouse carotid artery after surgery [38]. As shown in Fig. 2A, no apparent vascular remodeling was observed in the vessels of sham-operated mice, but the experimental groups of ligation, ligation with gel alone, and ligation with scrambled peptide, revealed significant neointimal formation. There was no difference among these three groups in terms of intima-media area (Intima + Media), area bound by the external elastic lamina (EEL) and intima/media ratio at 7 and 14 days post-ligation (Fig. 2A through 2E and Table 1), indicating the pluronic gel alone and the scrambled peptide have no effect on neointimal formation. Carotid arteries treated with R1R2 peptide, however, showed a dramatic attenuation in injury-induced neointimal formation (Fig. 2A through 2E). Morphometric analyses revealed significantly reduced intima-media areas (Intima + Media), EEL area and intimal/medial ratios 7 days and 14 days after artery ligation in R1R2-treated mice as compared to the control mice (Fig. 2C through 2E and Table 1). Interestingly, there was no significant decrease in the lumenal area in the R1R2-treated vessels at day 7 and 14 after injury when compared to the controls (Fig. 2B). Taken together, our study indicates that R1R2 treatment significantly attenuates neointimal formation in ligated vessels of mice.

**Table 1:** Morphometric analysis of vascular remodeling in experimental groups.

	Lumen (μm^2^)		Intima+Media (μm^2^)		EEL Area (μm^2^)		Intima/Media Ratio	
	7 day	14 day	7 day	14 day	7 day	14 day	7 day	14 day
Sham	18298 ± 4314 (n = 6)	18517 ± 2047 (n = 7)	20851 ± 1166 (n = 6)	22044 ± 1397 (n = 7)	53213 ± 14664 (n = 6)	41749 ± 2474 (n = 7)	0 (n = 6)	0 (n = 7)
Ligation	49572 ± 2566 (n = 4)	32383 ± 7037 (n = 7)	47420 ± 2489 (n = 4)	64621 ± 13716 (n = 7)	96993 ± 4454 (n = 4)	97004 ± 17488 (n = 7)	0.1559 ± 0.0250 (n = 4)	0.5426 ± 0.1848 (n = 7)
Ligation + Gel	42024 ± 4161 (n = 9)	32779 ± 9855 (n = 7)	42586 ± 2919 (n = 9)	59264 ± 7374 (n = 7)	84610 ± 6072 (n = 9)	99093 ± 14923 (n = 7)	0.2074 ± 0.0414 (n = 9)	0.4972 ± 0.1438 (n = 7)
Ligation + Scrambled Peptide	44901 ± 4552 (n = 4)	37551 ± 11976 (n = 4)	47803 ± 1854 (n = 4)	65441 ± 11973 (n = 4)	91403 ± 3805 (n = 4)	92240 ± 13572 (n = 4)	0.1807 ± 0.060(n = 4)	0.4754 ± 0.1143 (n = 4)
Ligation + R1R2	32038 ± 2870 (n = 10)	25776 ± 4286 (n = 9)	29208 ± 884 (n = 10)	25706 ± 1220 (n = 9)	64905 ± 3680 (n = 10)	51482 ± 4981 (n = 9)	0.0424 ± 0.0133 (n = 10)	0.0709 ± 0.0231 (n = 9)

### R1R2 Decreases Extracellular Matrix Deposition after Vascular Injury

**Fig 3:**
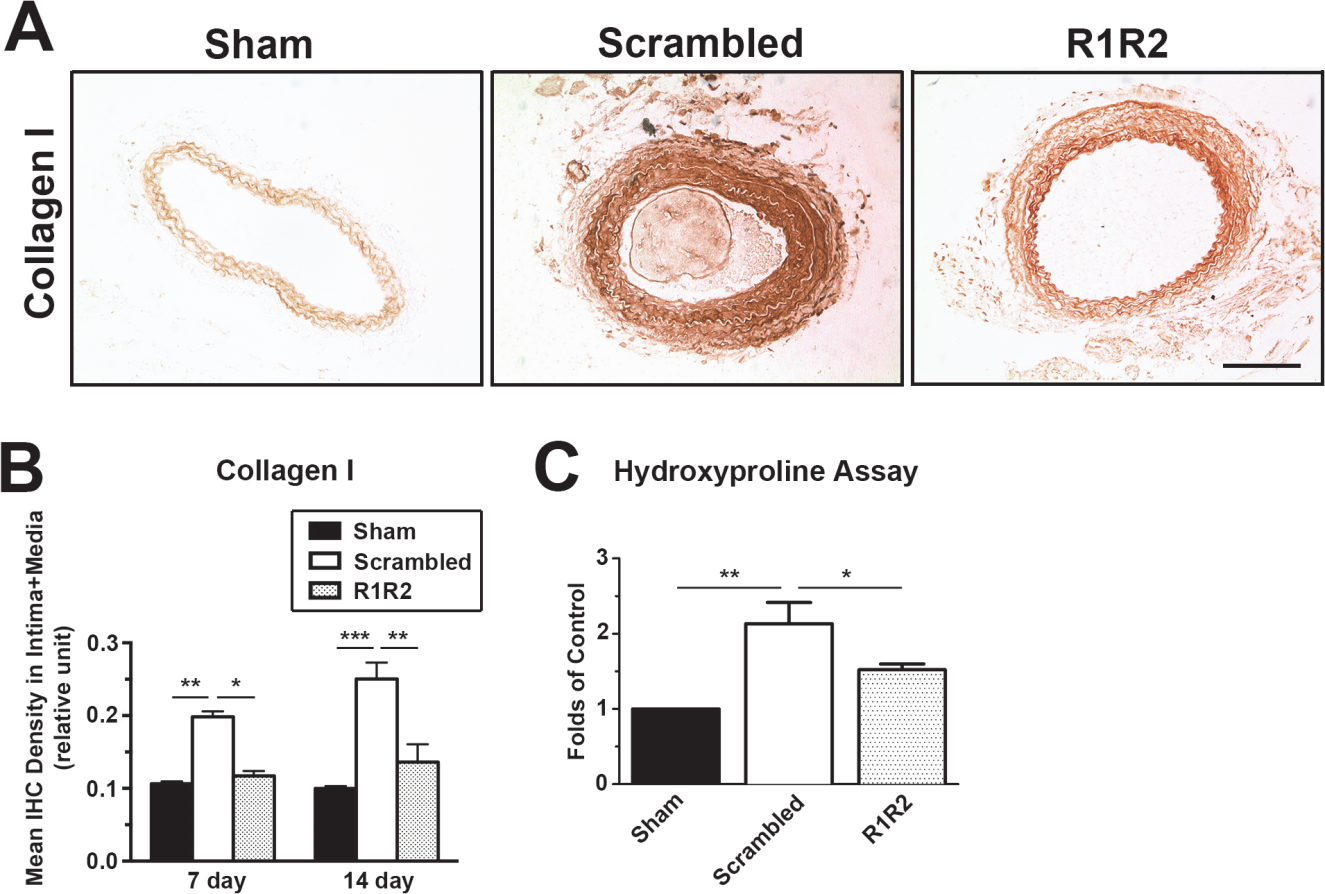
R1R2 decreases collagen content in the ligated vessel. (A) Immunohistochemistry (IHC) for collagen type I 14 days post-surgery. Bar, 100 μm. (B) Quantification of immunostaining intensity of collagen I in the intima-media area 7 (Sham: n = 6, Scrambled: n = 6, R1R2: n = 6) and 14 days (Sham: n = 6, Scrambled: n = 6, R1R2: n = 5) after the ligation. (C) Collagen content was biochemically measured by hydroxyproline assay in the carotid artery 14 days after ligation. n = 3 for each experimental group. * indicates p<0.05, ** p< 0.01 and *** *p*< 0.001.

**Fig 4:**
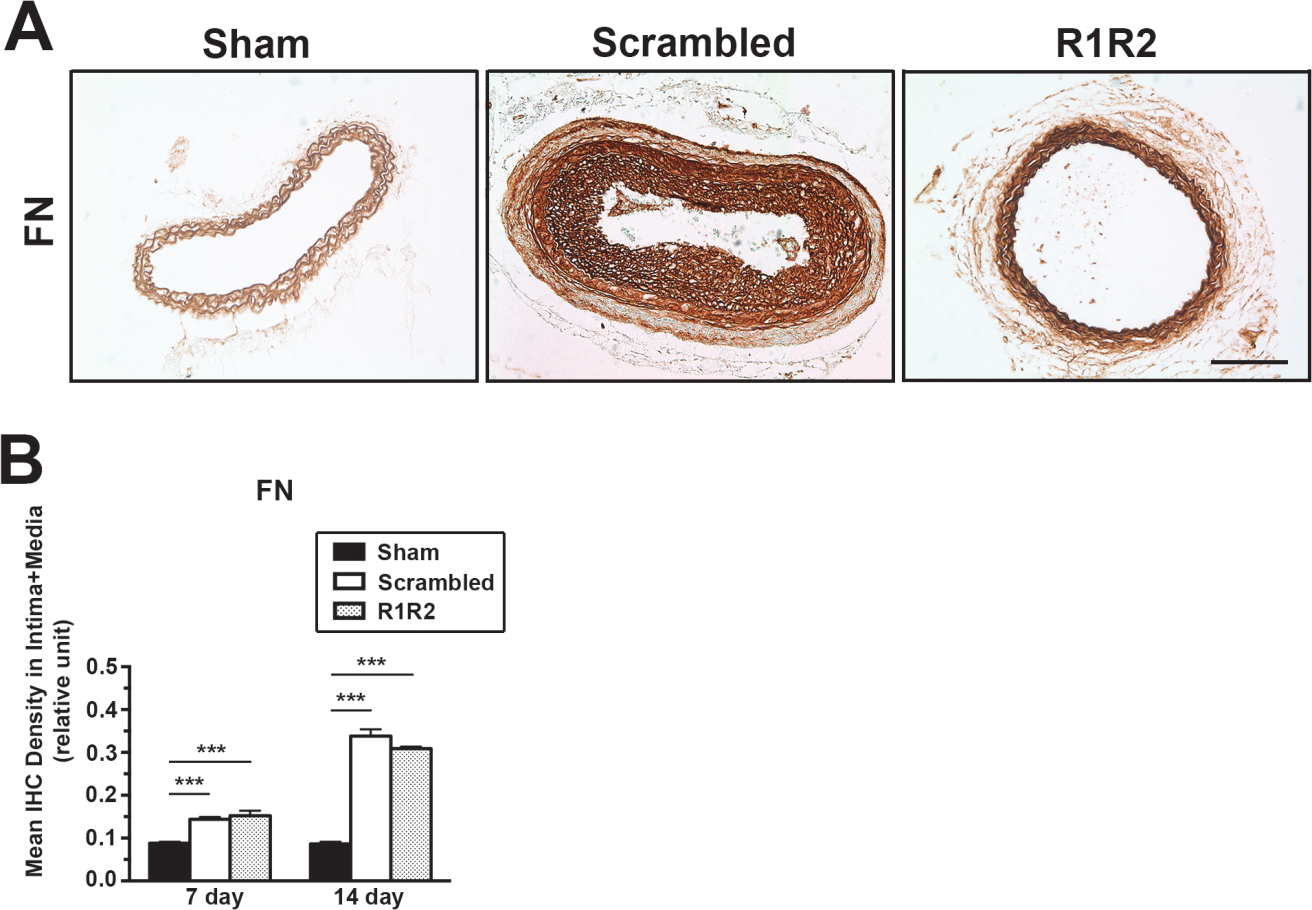
R1R2 does not attenuate FN content in the left carotid artery. (A) IHC for FN 14 days post-surgery. Bar, 100 μm. (B) Quantification of IHC intensity of FN in the intima-media area 7 (Sham: n = 6, Scrambled: n = 6, R1R2: n = 6) and 14 days (Sham: n = 7, Scrambled: n = 7, R1R2: n = 7) after ligating the vessel. * indicates p<0.05, ** p< 0.01 and *** *p*< 0.001.

Our data show that R1R2 peptide inhibits the binding of collagen to FN (Fig. 1), which is consistent with published data in vitro [29,30]. To determine the inhibitory effect of R1R2 on ECM accumulation in vessels, we performed immunohistochemistry (IHC) and hydroxyproline assay for collagen type I. As aforementioned, no apparent difference in morphometry was observed among the groups of ligation, ligation with gel alone, and ligation with scrambled peptide (Fig. 2). Therefore, we used the experimental group treated with scrambled peptide as the control group. In Fig. 3A and 3B, IHC analysis showed a significant decrease of collagen type I intensity in R1R2-treated arteries compared to the control group on 7 and 14 days after the surgery (7 days: 0.117 ± 0.007, n = 6 vs. 0.198 ± 0.007, n = 6. 14 days: 0.136 ± 0.024, n = 5 vs. 0.151 ± 0.022, n = 6). Hydroxyproline assay (Fig. 3C, 1.522 ± 0.077, n = 3 vs. 2.133 ± 0.284, n = 3) showed a consistent reduction of collagen content in carotid vessels 14 days post-ligation. Previous in vitro studies have revealed that R1R2 inhibits the interaction between FN and collagen but does not affect FN fibril deposition into the ECM [29]. To determine whether R1R2 impairs FN accumulation in the vessel wall, we evaluated FN levels in the vessel wall after ligation using IHC (Fig. 4). Consistent with published in vitro results, there was no significant decrease of FN intensity between vessels treated with R1R2 and treated with scrambled peptide (7 days, 0.153 ± 0.012, n = 6 vs. 0.144 ± 0.005, n = 6. 14 days: 0.309 ± 0.005, n = 7 vs. 0.338 ± 0.016, n = 7). Collectively, these data show that R1R2 treatment in injured vessels significantly limits collagen type I deposition (Fig. 3), but not FN deposition (Fig. 4) 7 days and 14 days after ligation.

### R1R2 Prevents Smooth Muscle Cell Dedifferentiation, Proliferation and Migration

**Fig 5:**
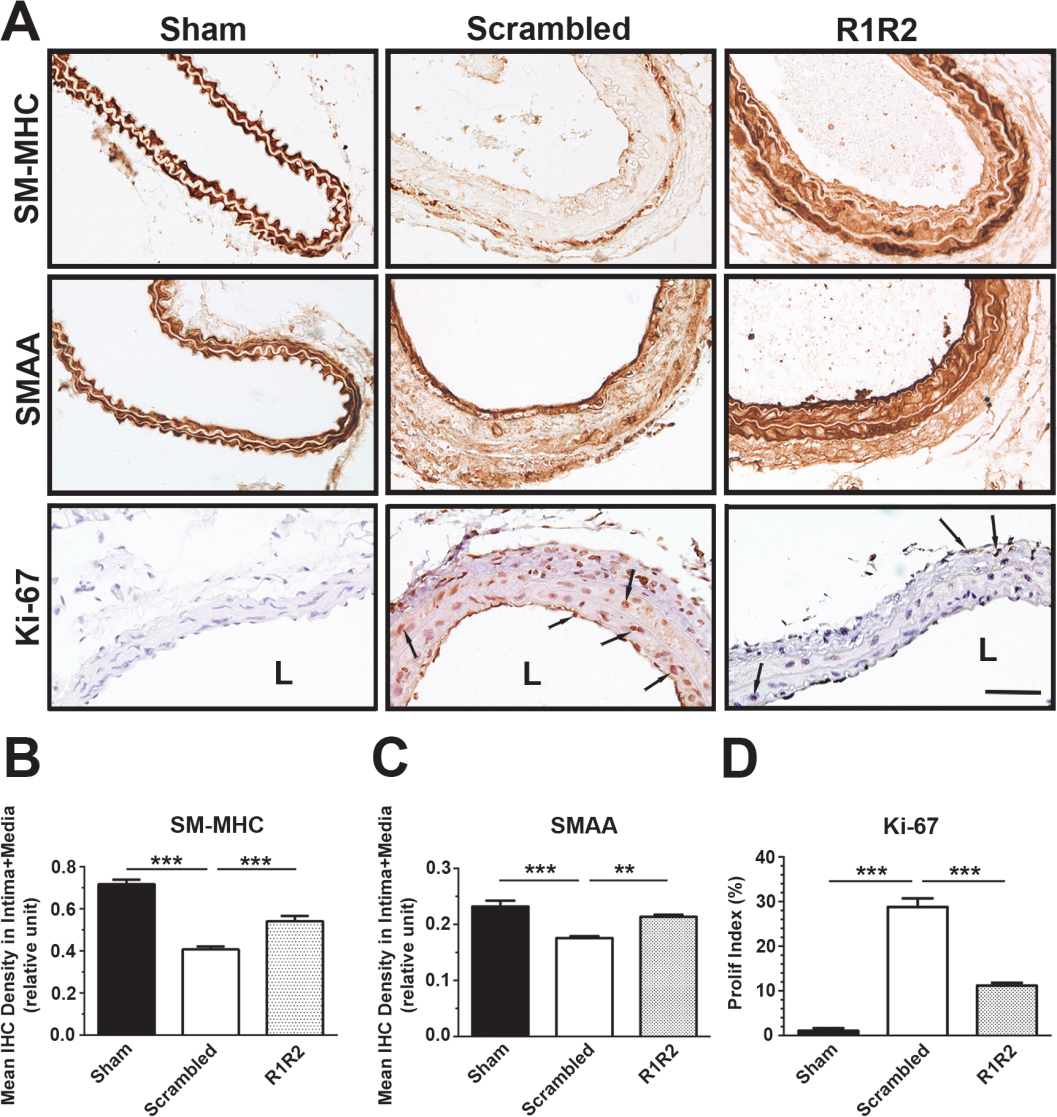
R1R2 maintains the contractile phenotype in smooth muscle cells. (A) Sections of the left carotid artery from mice 7 days post-surgery were immunostained for SM myosin heavy chain (SM-MHC) and SM α-actin (SMAA) and Ki-67. Arrows indicate Ki-67 (+) cells. Bar, 50 μm. (B) Quantification of IHC intensity of SM-MHC in the intima-media area (Sham: n = 6, Scrambled: n = 8, R1R2: n = 7). (C) Quantitative analysis of immunostaining intensities of SMAA in the intima-media area 7 days post-ligation (Sham: n = 5, Scrambled: n = 6, R1R2: n = 5). (D) Proliferation index (Ki-67 (+) cells/total cells) in the intima and media of the vessel wall (Sham: n = 6, Scrambled: n = 5, R1R2: n = 5). * indicates p<0.05, *** p< 0.001.

Leukocyte infiltration through the activated endothelium and phenotypic modulation of vascular smooth muscle cells are two hallmarks of vascular remodeling [41–43]. To further define the mechanism by which R1R2 regulates vascular remodeling, we investigated the effect of R1R2 on phenotypic modulation and leukocyte infiltration in the vessel wall. To assess the effect of R1R2 on smooth muscle phenotype, we immunostained sections of vessels 7 days post-ligation with the smooth muscle cell differentiation markers, myosin heavy chain (SM-MHC) and smooth muscle α-actin (SMAA) (Fig. 5A, 5B, and 5C). IHC analysis demonstrated that vessels treated with scrambled peptide showed significantly decreased SM-MHC (0.407 ± 0.0135, n = 8 vs. 0.717 ± 0.021, n = 6) and SMAA staining in the intima-media area (0.176 ± 0.004, n = 6 vs. 0.232 ± 0.011, n = 5). In contrast, vessels treated with R1R2 maintained the same high SM-MHC (0.541 ± 0.025, n = 7 vs. 0.407 ± 0.014, n = 8) and SMAA staining as the sham group (0.214 ± 0.004, n = 5 vs. 0.232 ± 0.011, n = 5). We also evaluated smooth muscle cell proliferation in the intima and media by performing IHC with Ki-67 (Fig. 5D). R1R2 treatment inhibited cell proliferation in the ligated artery compared to the scrambled peptide treatment (11.20 ± 0.62%, n = 5 vs. 28.77 ± 1.94%, n = 5).

**Fig 6:**
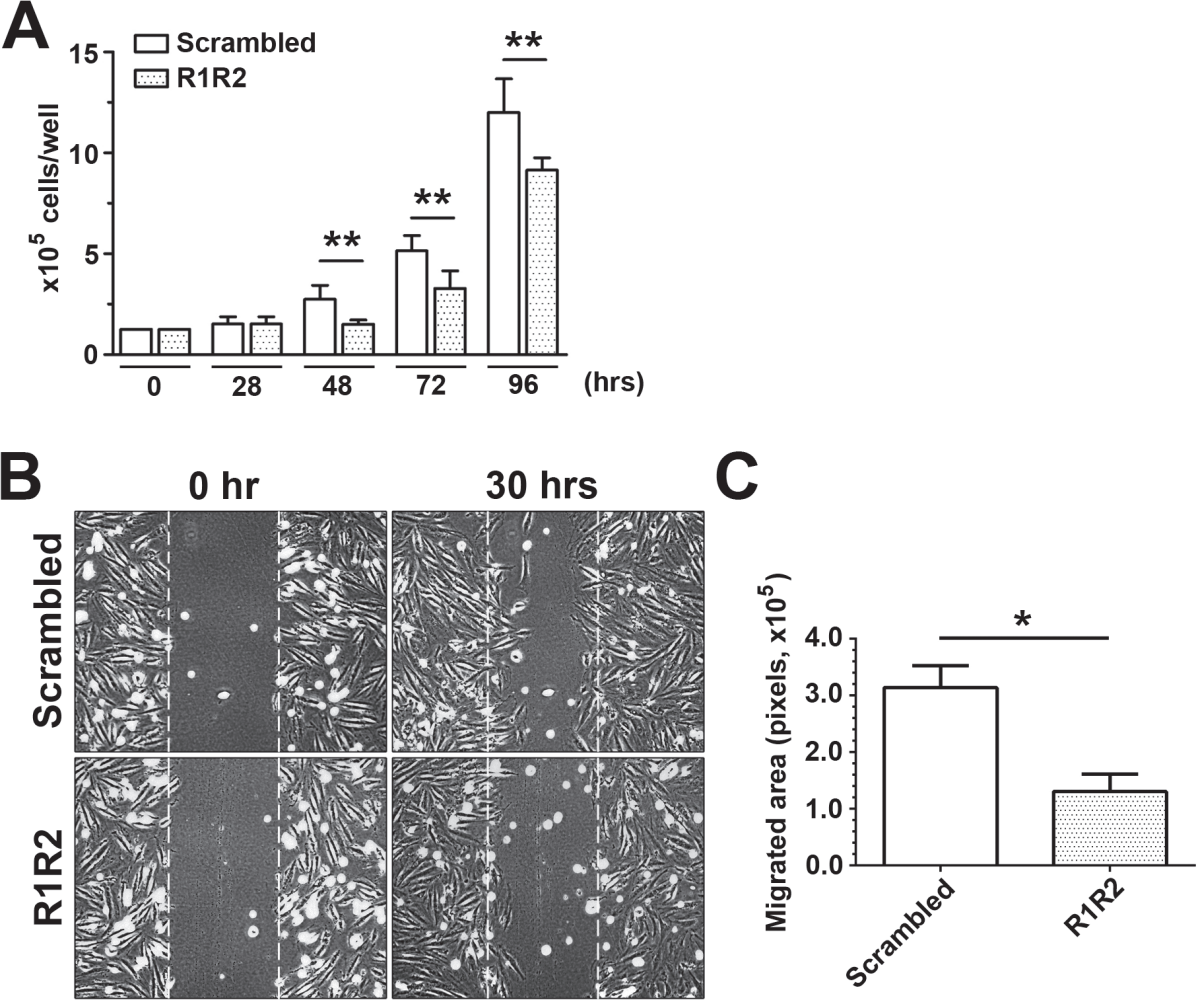
R1R2 reduces SMC proliferation and migration. (A) A7r5 SMC growth study over 96 hours. After serum starvation and treatment with R1R2 or scrambled peptides for 36 hours, cells were stimulated with 10% FBS over 96 hours. Cells were counted at the indicated time in the figure (n = 5). (B) A7r5 SMCs were pretreated with R1R2 or scramble peptide for 36 hours. SMCs underwent a scratch wound injury and were then stimulated with 10 ng/ml PDGF-BB for 30 hours. Representative images from the scratch wound assays are shown. (C) Quantitation of SMC migration in the scratch wound healing assay was performed by subtracting the cell-free area 30 hours after PDGF-BB stimulation from the cell-free area in the beginning (n = 5). * indicates p<0.05 and ** p< 0.01.

SMC proliferation and migration contribute to intima and media thickening after arterial injury [44,45]. Therefore, we evaluated the effect of R1R2 on SMC proliferation and migration in vitro. R1R2 peptide significantly inhibited A7r5 SMC growth after 48–96 hours in comparison with the scrambled peptide (1.5 ± 0.224 x 10^5^, n = 5 vs. 2.740 ± 0.688 x 10^5^, n = 5 at 48hr)(Fig. 6A). To determine whether R1R2 affects SMC migration, a wound healing assay was performed in SMCs stimulated with PDGF-BB. As shown in Fig. 6B, cells migrated more slowly in the presence of R1R2 than in the presence of scrambled peptide 30 hours post PDGF-stimulation. A quantitative analysis of SMC migration showed that R1R2 significantly reduced SMC migration in comparison with the scrambled peptide (130149 ± 53295, n = 3 vs. 313537 ± 66925, n = 3)(Fig. 6C). Together, these data show that R1R2 inhibits smooth muscle cell phenotype modulation, proliferation, and migration.

### R1R2 Inhibits Leukocyte Adhesion, Infiltration and CAM Expression

**Fig 7:**
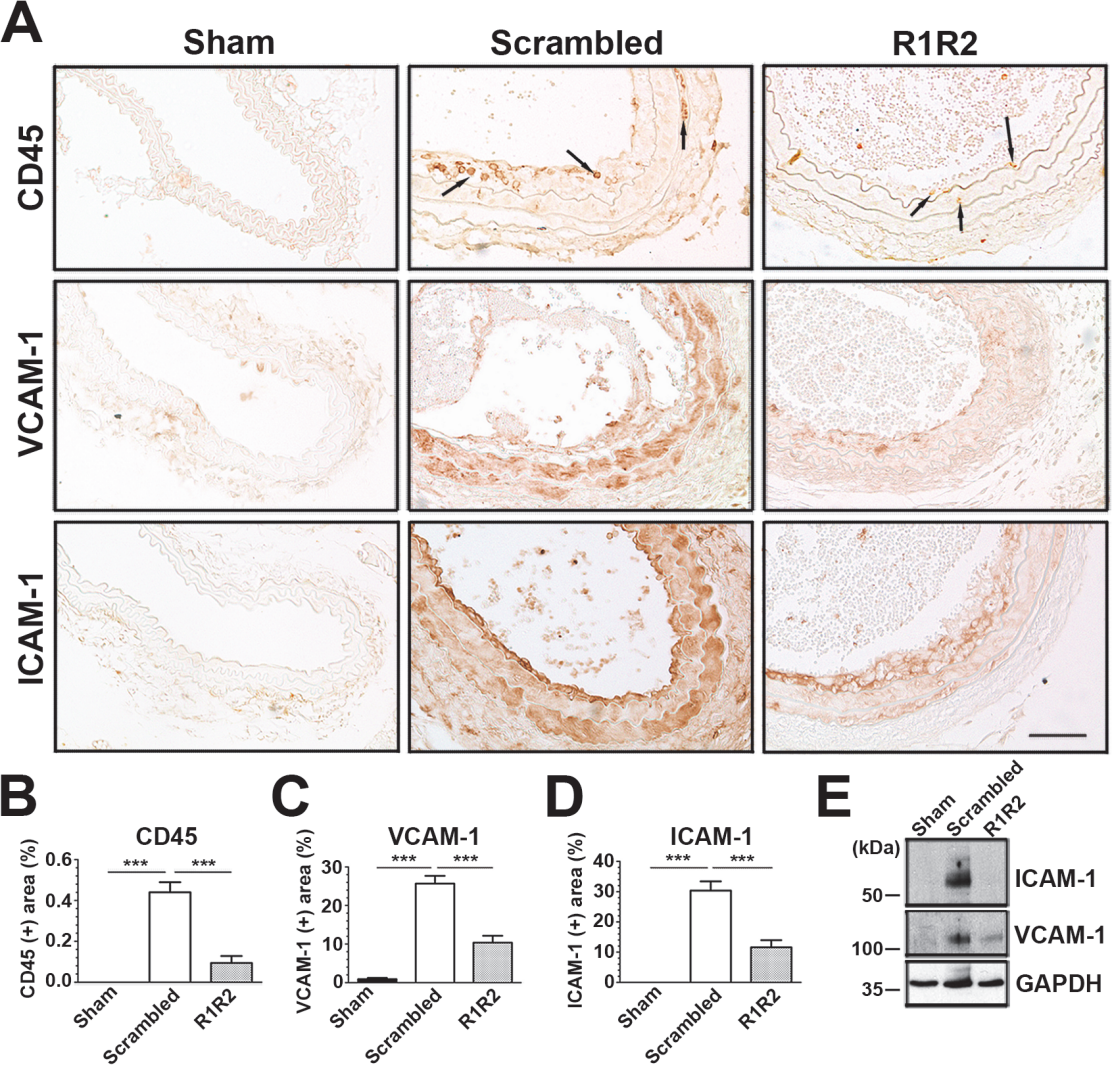
R1R2 decreases inflammatory cell accumulation and VCAM-1 and ICAM-1 levels. (A) Photographs showing representative immunostaining of CD-45, VCAM-1 and ICAM-1 in the left carotid artery from the animals subjected to ligation 7 days after the surgery. Arrows indicate CD45 (+) leukocytes. Bar, 50 μm. (B-D) Percentage of the area which is CD45 (+) (Sham: n = 7, Scrambled: n = 6, R1R2: n = 5) (B), VCAM-1 (+) (Sham: n = 7, Scrambled: n = 9, R1R2: n = 7) (C) or ICAM-1 (+) (Sham: n = 5, Scrambled: n = 8, R1R2: n = 7) (D) were assessed in the intima-media of the vessel. (E) Western blot analysis of ICAM-1 and VCAM-1 expression in ligated carotid artery at 7 days. Equal protein loading was confirmed with GAPDH. * indicates p<0.05 and ** p<0.01.

Inflammatory cell infiltration is a key event in neointimal formation after vascular injury [42,46]. Consistent with the data shown by other labs [35], reduced flow induced an increase of CD45(+) leukocytes in the intima and media 7 days post-surgery (Fig. 7). Of note, we observed a dramatic decrease in the number of CD45 (+) cells in response to R1R2 treatment (0.094 ± 0.035%, n = 5 vs. 0.441 ± 0.049%, n = 6). Furthermore, R1R2 also decreased the expression of cell adhesion molecules. IHC analysis showed that R1R2 application resulted in a dramatic reduction of vascular cell adhesion molecule-1 (VCAM-1) (Fig. 7A and 7C, 10.36 ± 1.802%, n = 7 vs. 25.72 ± 1.966%, n = 9) and intercellular cell adhesion molecule-1 (ICAM-1) (Fig. 7A and 7D, 11.58 ± 2.387%, n = 7 vs. 30.39 ± 3.016%, n = 8) expression in comparison with control peptide-treated vessels 7 days after ligation. Additionally, immunoblotting of VCAM-1 and ICAM-1 also showed a similar reduction in protein levels in the carotid artery following surgery (Fig. 7E). Taken in aggregate, our in vivo findings uncover an inhibitory effect of R1R2 peptide on leukocyte infiltration and CAM expression in the arterial wall after injury.

**Fig 8:**
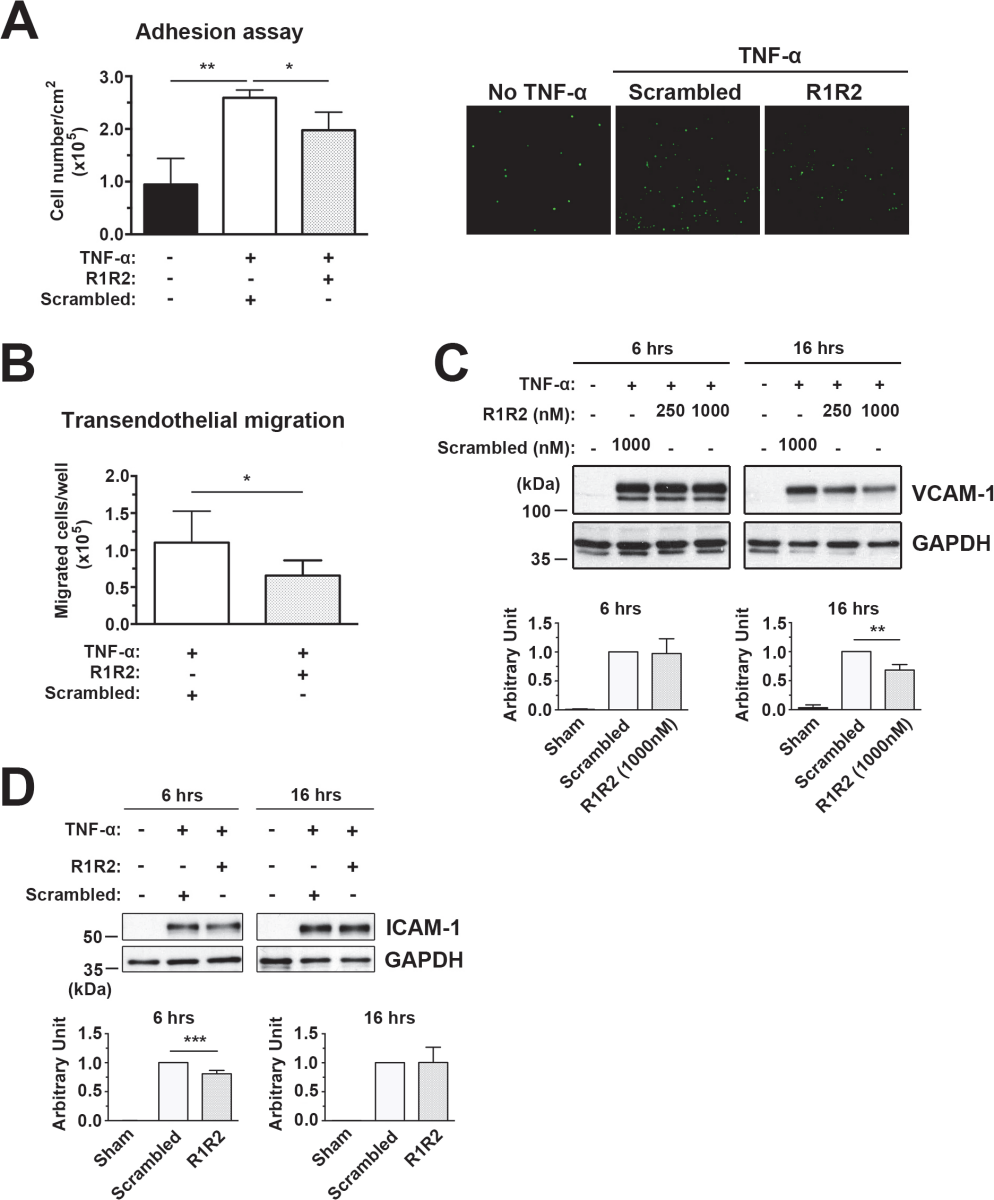
R1R2 decreases TNF-α-induced monocyte U937 cell adhesion to HUVECs and transendothelial migration and reduces ICAM-1 and VCAM-1 levels. (A) HUVECs were pretreated with R1R2 or scramble peptide before treatment with TNF-α (10ng/ml) for 6 hours in the continued presence of R1R2 or scrambled peptide. Calcein-AM labeled U937 monocyte adhesion to TNF-α HUVECs was quantitated by fluorescence intensity. Microscopic images showing U937 monocytes adhering to HUVECs as assessed by in vitro adhesion assay. (B) Calcein-AM-labeled U937 monocytes transmigrated through TNF-α-treated HUVECs. (C) Western blot analysis of ICAM-1 and VCAM-1 expression in TNF-α-treated HUVECs. * indicates *p*<0.05 and ** *p*< 0.01.

In injured vessels, there is increased expression of cell adhesion molecules in the activated endothelium, leading to enhanced leukocyte recruitment and transendothelial infiltration [47,48]. Our study demonstrates that there is a reduction in leukocyte infiltration in the vessel wall in R1R2-treated animals compared to scrambled peptide-treated mice (Fig. 7). To determine whether R1R2 modulates leukocyte recruitment to activated endothelial cells during inflammation, we assessed monocyte adhesion to HUVECs and transendothelial migration, which both involve interaction with cell adhesion molecules such as ICAM-1 and VCAM-1. In the adhesion assay, HUVECs treated with 10 ng/ml TNF-α for 16 hours were incubated with calcein-AM stained monocyte U937 cells. As shown in Fig. 8A, TNF-α-induced monocyte U937 adhesion to HUVECs was significantly blunted by the R1R2 peptide (197697 ± 34184 vs. 259093 ± 14720). In addition, R1R2 significantly reduced monocyte transendothelial migration in comparison to control peptide (Fig. 8B, 65633 ± 20412 vs. 110055 ± 42517). To rule out the possibility that the inhibitory effect of R1R2 on U937 adhesion and transmigration was due to a decrease in endothelial cell number, we assayed the effect of R1R2 on cell proliferation in comparison with control peptide-treated cells. No significant decrease was observed between the R1R2-treated HUVECs and the control group at the start of the adhesion assay (data not shown). Consistent with our in vivo data (Fig. 7), the expression levels of ICAM-1 and VCAM-1 on HUVECs were significantly decreased with R1R2 treatment (Fig. 8C and 8D). Interestingly, the inhibitory effect of R1R2 on ICAM-1 expression was transient and detected earlier than the effect on VCAM-1 expression after TNF-α stimulation (Fig. 8D). This result is supported by a previous report showing that VCAM-1 expression is induced by ICAM-1 activation or clustering [49]. Taken together, these data show that R1R2 reduces monocyte adhesion and infiltration, as well as the expression of cell adhesion molecules by endothelial cells.

## Discussion

In this study, we showed that the collagen inhibitory peptide, R1R2, is an effective inhibitor of neointimal formation induced by reduced blood flow (Fig. 2). Consistent with published in vitro data [29,30], our in vivo data demonstrate that R1R2 prevents collagen type I deposition in the ECM of the vessel wall after vascular injury (Fig. 3). R1R2 treatment maintains smooth muscle cells in the differentiated state (Fig. 5), and dramatically inhibits inflammatory cell infiltration into the vessel wall, with lower levels of ICAM and VCAM expression in the intima and media where SMCs are highly proliferative and migratory during the early stages of vascular remodeling (Fig. 7). Further, our in vitro studies demonstrate that R1R2 reduces cultured SMC proliferation and migration (Fig. 6), and also decreases monocyte adhesion and transmigration through endothelial cells (Fig. 8).

Pharmacological inhibition of collagen synthesis is known to decrease fibrotic diseases and improve organ function [50–52]. In this study, we demonstrated that R1R2 efficiently decreases collagen I deposition in the vessel wall. The compliance of the vessel wall is dependent on the relative content of two major proteins: collagen and elastin. Excess deposition of collagen is known to result in arterial stiffness [53–56]. It is well documented that elevated arterial stiffness is highly associated with atherosclerosis [57–59], and arterial stiffness is regarded as a useful marker of the extent of atherosclerosis in the aorta [60]. Hence, the ability of the R1R2 peptide to decrease collagen deposition is likely to normalize arterial wall stiffness and improve vascular function.

Activated endothelium and the subsequent inflammatory cell recruitment have been shown to be decisive players for SMC phenotypic modulation during the process of pathological vascular remodeling. Growth factors, cytokines and chemokines released by the activated endothelium and infiltrated inflammatory cells promote SMCs switching to the synthetic phenotype characterized by enhanced proliferating/migratory abilities and the expression of inflammatory transcriptional mediators, cell adhesion molecules, pro-inflammatory cytokines and chemokines [61–64], which further exacerbate the inflammatory responses in the pathological processes of the vascular remodeling. ECM proteins are also known to modulate proinflammatory gene expression in SMCs [65,66]. For examples, SMCs cultured on collagen type IV show reduced VCAM-1 expression in comparison with SMCs cultured on collagen type I [65]. The expression of cell adhesion molecules on SMCs is thought to facilitate the accumulation of infiltrated leukocytes in the vessel wall [67,68]. The in vivo data of this study revealed that R1R2 significantly reduced VCAM-1 and ICAM-1 expression in the intima and media of the vessel wall after injury, suggesting that R1R2 inhibits the inflammatory reactions induced by activated endothelium, and also blocks inflammation resulting from the activation of SMCs after vascular injury.

In vitro studies have shown that FN is required as a scaffold for collagen deposition [28,29,31]. Inhibiting FN polymerization with antibodies or inhibitors prevents FN and collagen fibril deposition into the ECM in vitro [27,31]. In our previous study, we examined the effect of pUR4, a recombinant peptide derived from the F1 adhesin that is a potent inhibitor of FN polymerization in vitro [69], on flow-induced vascular remodeling. Like the R1R2 peptide in this study, the pUR4 peptide also attenuates intima-media thickening in ligated mouse vessels, decreases SMC phenotypic modulation, inhibits cell adhesion molecule expression, and decreases inflammatory cell infiltration into the vessel wall [38]. pUR4 treatment also dramatically decreases the levels of FN and collagen type I in the vessel wall after surgery in mice [38]. In this study the R1R2 peptide is shown to prevent the accumulation of collagen type I in the ECM, but does not inhibit FN accumulation. These data are consistent with our published in vitro studies showing that inhibiting FN-collagen interaction with R1R2 does not alter FN fibril formation [29]. The deposition of FN has been shown to be important for the deposition of other ECM molecules, including collagen type I [27,70,71]. Therefore, our in vivo work to inhibit collagen type I deposition with R1R2 may provide an efficient treatment to attenuate neointimal formation without interfering with the levels of other ECM proteins in tissues.

Collagen type I can act as a signaling molecule by binding to cell surface receptors. In this study, we have shown that adventitial delivery of R1R2 attenuates smooth muscle cell activation and leukocyte infiltration. We postulate that R1R2 may decrease neointimal formation by inhibiting collagen type I fibril deposition, which uncouples collagen-collagen receptor signaling. Several collagen receptors have been identified. For instance, the collagen receptor UPARAP/Endo180 has been shown to be involved in collagen endocytosis [39,50,72,73]. Collagen-binding receptors including certain integrins and DDRs, physically connect cells to collagen, and mediate cellular responses to collagen [18–22]. Integrins are heterodimers composed of two distinct subunits, α and β. ECM ligands that bind to integrin receptors initiate integrin clustering and activation, which is followed by recruitment of focal adhesion kinase (FAK) to focal adhesion complexes, and autophosphorylation of FAK [74]. α1β1 and α2β1 are the major integrin receptors for collagen type I. Blocking β1 integrin-mediated signaling is known to inhibit smooth muscle cell proliferation, migration, and phenotypic transformation [75–77]. In addition, collagen-β1 integrin interactions are involved in cytokine secretion from cells [78,79], which promotes smooth muscle cell activation. α1β1 and α2β1 integrins have also been shown to play a crucial role in regulating inflammatory diseases [80]. Our data show that treatment of injured vessels with R1R2 decreases VCAM-1 and ICAM-1 expression in the intima and media. These results may be due to the blockade of integrin-mediated signaling by R1R2. Several reports have demonstrated that collagen-β1 integrin ligation augments ICAM-1 expression on cells in a FAK-dependent manner [81–83], and pharmacologically inhibiting FAK blocks TNF-α-induced VCAM-1 expression [84].

R1R2 may also decrease the interaction between collagen type I and DDRs, and interrupt DDR1 and DDR2-mediated signaling pathways [22]. DDRs are homodimers containing tyrosine kinase in their cytoplasmic domain [85,86]. Upon collagen binding, the receptor dimerizes and induces autophosphorylation of the cytoplasmic domains [87]. DDR1 is known to regulate smooth muscle cell proliferation and migration [22,88,89]. Additionally, DDR1 and DDR2 are activated by polymerized collagen, and overexpression of DDR1 and DDR2 increase matrix metalloproteinase activities [90]. In vivo evidence show that interruption of DDR-induced signaling pathways significantly decreases neointimal formation [88,91]. In our study, inhibiting FN-collagen binding and collagen type I deposition attenuates neointimal formation in a flow-reduced vascular remodeling model. Therefore, we speculate that inhibiting collagen type I deposition with R1R2 peptide may uncouple collagen-integrin or collagen-DDR interactions, which in turn decrease cellular responses in SMCs and endothelial cell during vascular remodeling.

It is also possible that collagen deposition affects vascular remodeling due to the ability of collagen and/or collagen binding macromolecules to sequester growth factors and cytokines in the ECM [92–94]. ECM bound cytokines and growth factors are known to influence various cellular functions including cell proliferation, differentiation, and survival [95,96]. Changes in collagen deposition are also likely to influence the migration of inflammatory cells [97,98] and the production and/or release of inflammatory cytokines [99]. Our data demonstrate that inhibiting collagen type I matrix deposition by periadventitial delivery of R1R2 can efficiently attenuate neointimal formation after injury. Furthermore, in addition to eliminating collagen type I-dependent SMC proliferation and migration, R1R2 also significantly abolishes the inflammatory responses in the vessel wall, which is likely due to the decrease in collagen type I-mediated VCAM-1 and ICAM-1 expression, and subsequent inflammatory cell infiltration. Our study suggests the possibility that R1R2 may have therapeutic potential for treating occlusive vascular diseases.
